# Developing Patient-Specific Statistical Reconstructions of Healthy Anatomical Structures to Improve Patient Outcomes

**DOI:** 10.3390/bioengineering10020123

**Published:** 2023-01-17

**Authors:** Matthew A. Wysocki, Steven A. Lewis, Scott T. Doyle

**Affiliations:** Department of Pathology and Anatomical Sciences, Jacobs School of Medicine and Biomedical Sciences, University at Buffalo, Buffalo, NY 14203-1121, USA

**Keywords:** joint replacement, patient-specific, personalized medicine, prosthetics, statistical shape modeling, surgery

## Abstract

There are still numerous problems with modern joint replacement prostheses, which negatively influence patient health and recovery. For example, it is especially important to avoid failures and complications following hip arthroplasty because the loss of hip joint function is commonly associated with increased demand on the healthcare system, reoperation, loss of independence, physical disability, and death. The current study uses hip arthroplasty as a model system to present a new strategy of computationally generating patient-specific statistical reconstructions of complete healthy anatomical structures from computed tomography (CT) scans of damaged anatomical structures. The 3D model morphological data were evaluated from damaged femurs repaired with prosthetic devices and the respective damaged femurs that had been restored using statistical reconstruction. The results from all morphological measurements (i.e., maximum femoral length, Hausdorff distance, femoral neck anteversion, length of rotational center divergence, and angle of inclination) indicated that the values of femurs repaired with traditional prostheses did not fall within the +/−3 standard deviations of the respective patient-specific healthy anatomical structures. These results demonstrate that there are quantitative differences in the morphology of femurs repaired with traditional prostheses and the morphology of patient-specific statistical reconstructions. This approach of generating patient-specific statistical reconstructions of healthy anatomical structures might help to inform prosthetic designs so that new prostheses more closely resemble natural healthy morphology and preserve biomechanical function. Additionally, the patient-specific statistical reconstructions of healthy anatomical structures might be valuable for surgeons in that prosthetic devices could be selected and positioned to more accurately restore natural biomechanical function. All in all, this contribution establishes the novel approach of generating patient-specific statistical reconstructions of healthy anatomical structures from the CT scans of individuals’ damaged anatomical structures to improve treatments and patient outcomes.

## 1. Introduction

Each year, there are over 1 million hip arthroplasties completed around the world, a number that is expected to double within two decades because of the aging global population [1,2]. Although hip arthroplasty generally has a high rate of success, failure of hip joint replacements can be highly detrimental for patients and may require revision surgeries that increase the burden on healthcare systems [2,3,4,5]. Primary hip replacement surgeries place considerable burdens on healthcare systems around the world, with annual costs that exceed 7 billion dollars [6]. Considering the consequences of hip joint replacement failure for patients, as well as the increased technical requirements and expense of revision hip arthroplasty, it is imperative to continue developing hip arthroplasty as new technologies become available [7].

Hip arthroplasty is carried out primarily to address pain and loss of function due to osteoarthritis, but patients also undergo hip joint replacement to treat fractures, avascular necrosis, dysplasia, Paget’s disease, and rheumatoid arthritis [6,8]. Hip joint replacement surgeries can be divided into hemiarthroplasty and total hip arthroplasty [8,9,10,11]. Hemiarthroplasty generally replaces the femoral head and neck and includes a femoral stem to offer support and anchorage [9]. Total hip arthroplasty replaces the head and neck of the femur, but it also involves surgically modifying the acetabulum of the os coxae [9]. Hip fracture is treated using total hip arthroplasty or hemiarthroplasty, whereas advanced osteoarthritis is primarily treated using total hip arthroplasty [9,12].

The earliest hip arthroplasty methods tended to be effective for short periods of time in terms of pain relief and function, but as time passed, they frequently resulted in negative outcomes due to chronic infections [10,13,14,15]. Subsequent designs inherently failed to function as ball-and-socket joints because the artificial femoral head did not fit securely within the artificial acetabular cup and due to the acetabular cup itself being subject to displacement. These features caused mechanical instability and loss of the patient’s mobility as fibrocartilaginous materials formed around the prosthesis [16]. Modern prostheses used in total hip arthroplasty follow the conceptual design established by Sir John Charnley (often referred to as low friction arthroplasty), which is made up of a femoral component with femoral head, femoral neck, and femoral stem, as well as an acetabular component consisting of a cup that is fixed to the os coxae using a cement; a design that has been in use for six decades [11].

Unfortunately, there are still numerous problems with hip arthroplasty, including infection, degradation, failure of the prosthesis, and various fractures [1,11]. Successful hip arthroplasty has innate challenges due to the fact that the functional aspects of the hip joint require withstanding body weight and being able to move in every plane [17]. Accordingly, one of the most significant problems with current hip prostheses is dislocation, an outcome that occurs in 0.5 to 10 percent of total hip arthroplasty cases and 6 percent of hemiarthroplasty cases [9,18,19]. The degradation of the prosthetic structure itself is another major problem. For example, linear wear can compromise the function of the hip prosthesis because the femoral head develops asymmetric positioning within the acetabular cup [9]. Furthermore, prostheses with polyethylene components encounter problems because the polyethylene debris particles cause periprosthetic osteolysis, a process that eventually leads to implant failure in which the prosthesis separates from the bone [11].

In addition to these negative outcomes for hip replacements, fractures can occur either in the bone or in the prosthesis. Fractures of the bone usually occur in the form of periprosthetic fractures along the femoral component of the prosthesis, rather than in the os coxae surrounding the acetabular cup [9,11]. Prosthetic fractures can vary according to material type. Ceramic hip prostheses offer the advantages of excellent biocompatibility and hardness, but the brittle nature of the material carries an increased risk of catastrophic fracture to the entire femoral head and/or neck [20,21]. In contrast, metal-fatigue stress fractures usually occur in the femoral stem or the femoral neck [9]. This fracture of the femoral stem is a problem that is severely worsened by the enormous challenge of having to extract the osseointegrated distal fragment of the prosthesis from the femur [22]. Of the myriad problems that can afflict patients after hip arthroplasty, the most common reasons for hip revision surgery are dislocation and mechanical loosening [23].

Selecting the best possible treatment for hip fracture and pathology is essential to avoid increased demand on the healthcare system, reoperation, loss of independence, physical disability, and death of the patient [12]. Previous studies have identified substantial limitations in the accuracy and effectiveness of hip replacements [1,9,11,23,24]. Recent research studies have utilized the computational simulation technique of finite element analysis to make new discoveries about the biomechanics of the hip joint, as well as about the wear and failure of hip joint prostheses [25,26,27,28,29,30,31,32,33,34,35,36]. Furthermore, a comprehensive review of computational simulation of the hip joint prosthesis has revealed that it is essential for research and prosthesis development to transition from using a standard "normal" condition to being informed by subject-specific information, which takes into consideration the unique anatomical characteristics of individuals [37].

The current study uses the hip prosthesis as a model system for demonstrating a new approach to generating patient-specific statistical reconstructions of the missing portions of damaged anatomical structures. It is hypothesized that morphological disparities will be evident between femurs with prostheses and the patient-specific statistical reconstructions of the healthy structure of those same femurs. Specifically, the current study establishes a novel computational approach to generating patient-specific statistical reconstructions of healthy anatomical structures from computed tomography (CT) scans of individuals’ damaged anatomical structures.

## 2. Materials and Methods

### 2.1. Anatomical Data Description

**Figure 1:**
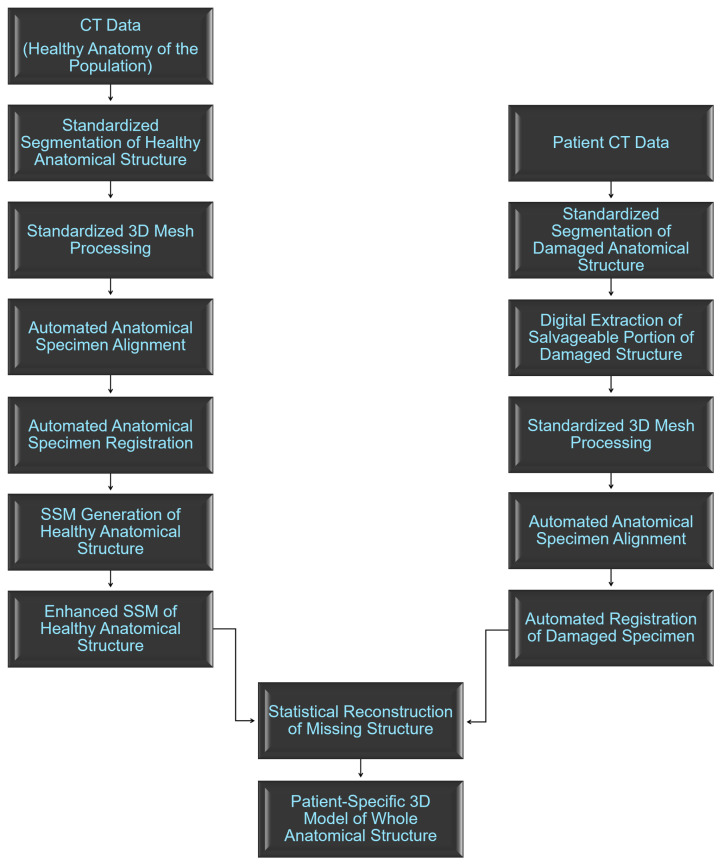
Overview of the process of generating patient-specific statistical reconstructions of healthy anatomical structures.

In order to be able to statistically reconstruct the missing portions of anatomy on a damaged structure (Figure 1), it was first necessary to generate a statistical shape model (SSM) of the healthy anatomical structure, which was generated using a left femur model system following the previously established protocol for obtaining SSMs from cadaveric CT data [38]. These cadaveric CT data were provided by the charitable donations of the Anatomical Gift Program of the Jacobs School of Medicine and Biomedical Sciences, University at Buffalo (UB). Each of the cadaveric donors provided informed consent prior to death, directing that their bodies following death were to be donated to the University at Buffalo, the State University of New York, as unrestricted gifts for the purposes of medical study and research.

For the healthy sample, cadaveric donors with joint replacements and surgical pins were excluded because of the artificial morphology of these structures and their associated CT data artifacts. In addition, individuals with pathologies such as osteoarthritis and osteoporosis were excluded from the sample used to generate the SSM of the healthy anatomical structure (*n* = 35). The donor cadavers were imaged prior to dissection using a GE Discovery 690 helical CT, which was carried out at the UB Clinical and Translational Research Center (CTRC).

### 2.2. Standardized Data Processing

The standardized segmentation of cadaveric CT data was carried out in order to extract the anatomical structure without the introduction of morphological artifacts and inaccuracies [39,40]. Specifically, the healthy left femur structures were segmented using 3D Slicer, an open-source software platform for image processing and 3D data visualization [41]. The segmentation of all specimens was completed using the Kittler–Illingworth minimum error thresholding algorithm, and the femur 3D anatomical models were exported as stereolithography (.stl) files [42].

The open-source software Meshmixer was used for standardized data processing to remove isolated pieces and create watertight 3D meshes, which excluded mesh artifacts [43]. As well, the 3D meshes were standardized to 10,000 triangular faces in MeshLab, an open-source software program for mesh processing [44]. This was accomplished using quadric-based edge collapse decimation, which is an approach that more effectively preserves mesh morphology [45,46]. The decimation parameters were set for the preservation of mesh boundaries, preservation of normals, and preservation of topology. Additionally, transformation alignment was applied to the 3D meshes in order to remove the spatial variation between the specimens that would otherwise negatively impact the accuracy of the morphology in the eventual SSM.

### 2.3. Anatomical Data from Damaged Structures

**Figure 2:**
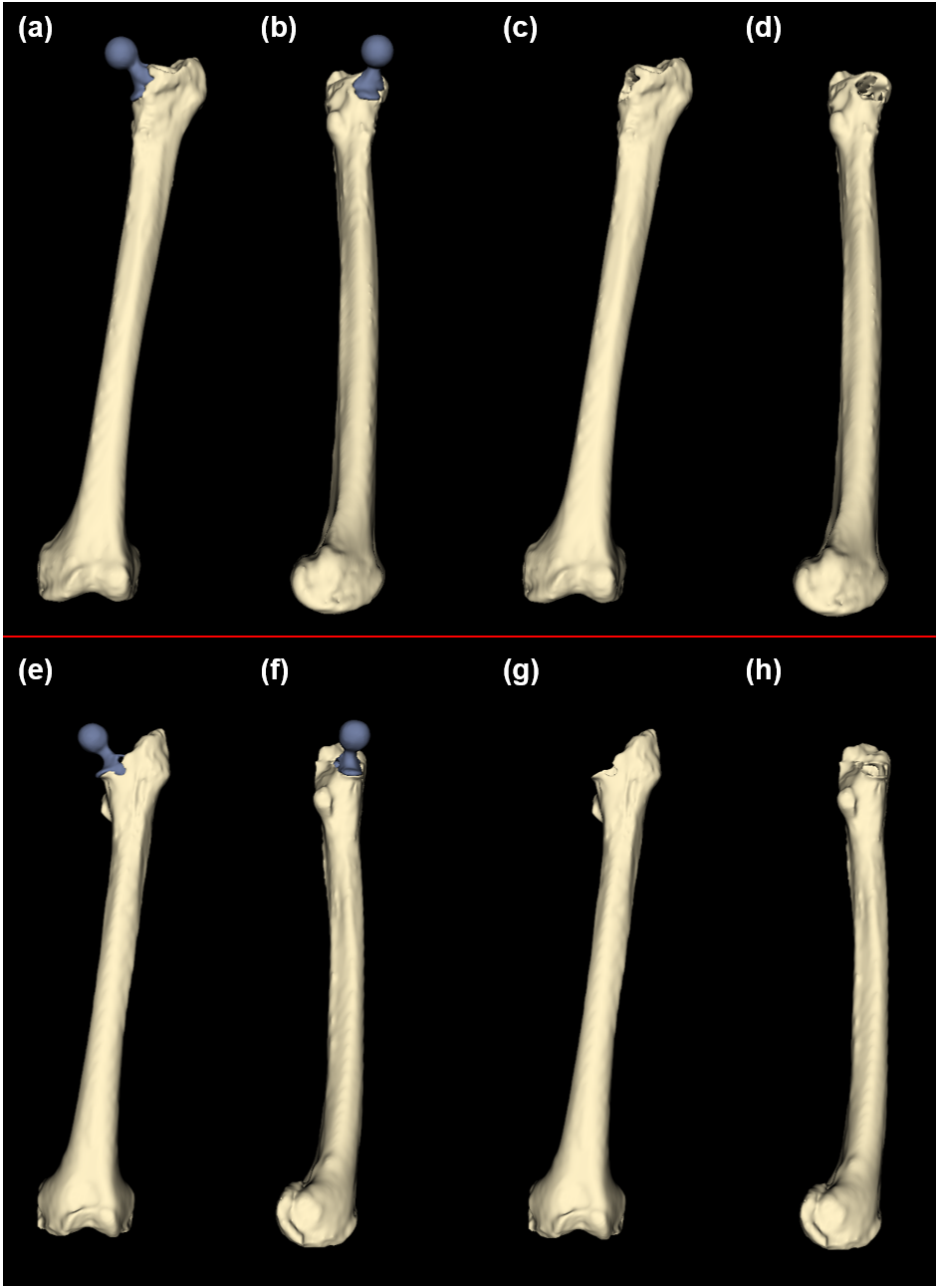
Damaged femur structure; PAS-W-SRA (**a**–**d**) and PAS-W-SRB (**e**–**f**): (**a**) damaged femur with traditional hip prosthesis, anterior view; (**b**) damaged femur with traditional hip prosthesis, medial view; (**c**) damaged femur 3D anatomical model only, anterior view; (**d**) damaged femur 3D anatomical model only, medial view; (**e**) damaged femur with traditional hip prosthesis, anterior view; (**f**) damaged femur with traditional hip prosthesis, medial view; (**g**) damaged femur 3D anatomical model only, anterior view; (**h**) damaged femur 3D anatomical model only, medial view. Bone is shown in beige, and prostheses are shown in gray.

The statistical reconstruction process was tested using the left femur anatomical structure from the remaining osteological portion of the femurs from individuals who had been treated with arthroplasty. Instances of damaged anatomical structures were used in order to assess the potential for the statistical reconstruction of anatomy to improve upon the existing methods for treating anatomical structures afflicted by injury or pathology (Figure 2). The damaged femur specimens were both from the same morphological design of the total hip arthroplasty prosthesis comprising a metallic femoral head, neck, and anchor; a non-metallic liner; and an acetabular cup.

The segmentation of the damaged femur structure was carried out using the same procedure used for all the healthy femur specimens with the Kittler–Illingworth minimum error thresholding algorithm [42]. The metallic femur prosthesis of each specimen was separately extracted using a narrow, high-intensity threshold for segmentation. The associated damaged femur osteological structure and the prosthesis 3D meshes were subjected to the aforementioned standardized data-processing steps used for the healthy anatomical structure data.

### 2.4. Statistical Shape Model Generation

**Figure 3:**
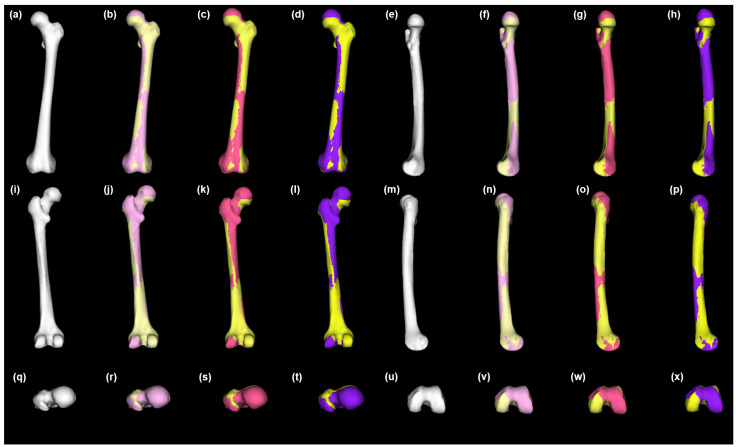
Healthy left femur statistical shape model and variation across the population. Anterior view (**a**–**d**), medial view (**e**–**h**), posterior view (**i**–**l**), lateral view = (**m**–**p**), superior view = (**q**–**t**), and inferior view (**u**–**x**). Mean = white (**a**,**e**,**i**,**m**,**q**,**u**); +1 SD = pink (**b**,**f**,**j**,**n**,**r**,**v**); −1 SD = pastel yellow (**b**,**f**,**j**,**n**,**r**,**v**); +2 SD = magenta (**c**,**g**,**k**,**o**,**s**,**w**); −2 SD = lemon chiffon (**c,g,k,o,s,w**); +3 SD = purple (**d**,**h**,**l**,**p**,**t**,**x**); −3 SD = yellow (**d**,**h**,**l**,**p**,**t**,**x**). SD = standard deviation.

These standardized 3D anatomical models of the healthy femur structure were used to generate an SSM from the population using the programming language Scala [47,48]. Although most of the spatial variation between the specimens was already removed during the aforementioned standardized data processing, an iterative closest-point (ICP) algorithm was used to rigidly align all the 3D anatomical models in the sample. The process removed any remaining spatial variations between the 3D anatomical models due to translational and rotational disparities so that the SSM only captured the morphological variation. This automated rigid alignment process was conducted using 50 evenly distributed pseudo-landmarks and 150 iterations per specimen. The high quality of the 3D meshes in the sample permitted the utilization of the automated processing of the specimens via non-rigid parametric registration, using 1000 points per mesh and 100 iterations. Finally, principal component analysis (PCA) was carried out to yield the SSM (Figure 3).

In addition, the SSM was further improved to model the healthy femur structure. In particular, the model was enhanced using a Gaussian process (GP) with a symmetric Gaussian kernel, which is a procedure that improves the SSM by making it more capable of capturing all the shape variations in the healthy structure. Furthermore, this process was carried out because augmentation helps any given SSM to be resistant to any potential remaining noise or errors in the dataset. This enhanced SSM of the healthy femur structure was then used to statistically reconstruct the damaged femur structures.

### 2.5. Statistical Reconstruction of Healthy Anatomy

Patient-specific statistical reconstructions were obtained for each of the damaged femurs. This was carried out using the damaged femur structure without the metallic prosthesis, enhanced SSM, and GP regression for model fitting. For a particular specimen, this process yielded a complete healthy femur structure that maintained the morphology of the part of the anatomy that was still intact and the corresponding statistical reconstruction of the healthy anatomy that had been absent.

Moreover, the statistical reconstruction process was applied to obtain the different statistically determined variations of the restored healthy anatomical structure. For each damaged specimen, 7 statistically reconstructed 3D anatomical model variants were generated for the quantitative evaluation of morphology. These variants based on the SSM of the healthy population included the statistical reconstruction mean anatomical structure, +1 standard deviation (SD) statistical reconstruction anatomical structure, −1 SD statistical reconstruction anatomical structure, +2 SD statistical reconstruction anatomical structure, −2 SD statistical reconstruction anatomical structure, +3 SD statistical reconstruction anatomical structure, and −3 SD statistical reconstruction anatomical structure.

### 2.6. Morphological Data Collection

**Figure 4:**
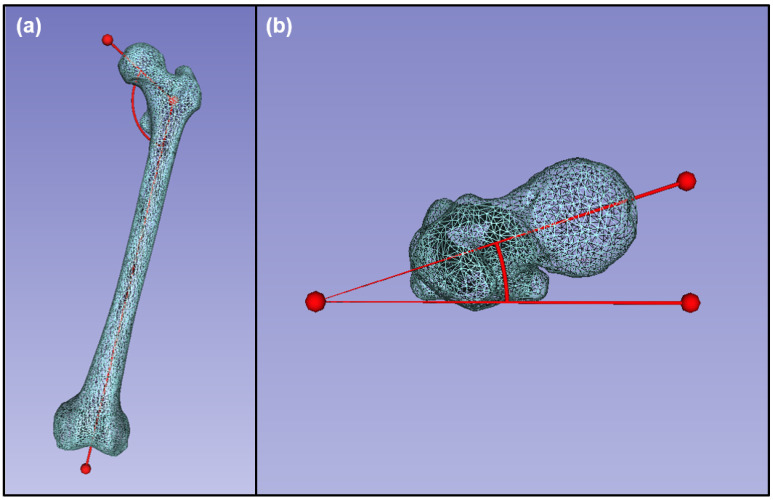
Morphological data collected from an example healthy femur: (**a**) angle of inclination, anterior view; (**b**) femoral neck anteversion, superior view.

To evaluate how the traditional femur prosthetic compares with the patient-specific statistically reconstructed healthy anatomy, quantitative morphological data were collected from the traditional prosthesis plus the damaged femur 3D anatomical model as well as the 7 statistically reconstructed 3D anatomical models. Morphological data consisted of commonly used anatomical measurements for the femur (Figure 4). These data were collected using 3D Slicer open-source software [41].

Five types of quantitative data were collected including the maximum femoral length, the Hausdorff distance, the femoral neck anteversion, the length of the rotational center divergence, and the angle of inclination. The maximum femoral length was defined as the maximum length between the femoral head and the farthest femoral condyle [49,50]. The Hausdorff distance was calculated using the mean statistical reconstruction of the healthy anatomical structure and the given anatomical model, which was measured for each of the specimens. The femoral neck anteversion (FNA), sometimes referred to as femoral torsion, was defined as the angle between the longitudinal axis of the head and neck of the femur and the axial plane that includes the most posterior points of the femoral condyles and the most posterior point of the greater trochanter [50]. The length of the rotational center divergence was defined as the length between the center of rotation of the femoral head of the statistical reconstruction mean anatomical structure and the center of rotation of the femoral head of the structure being measured. The angle of inclination, otherwise known as the neck-shaft angle (NSA), was defined as the angle between the longitudinal axis of the femoral head and neck and the longitudinal axis of the femoral shaft.

## 3. Results

**Figure 5:**
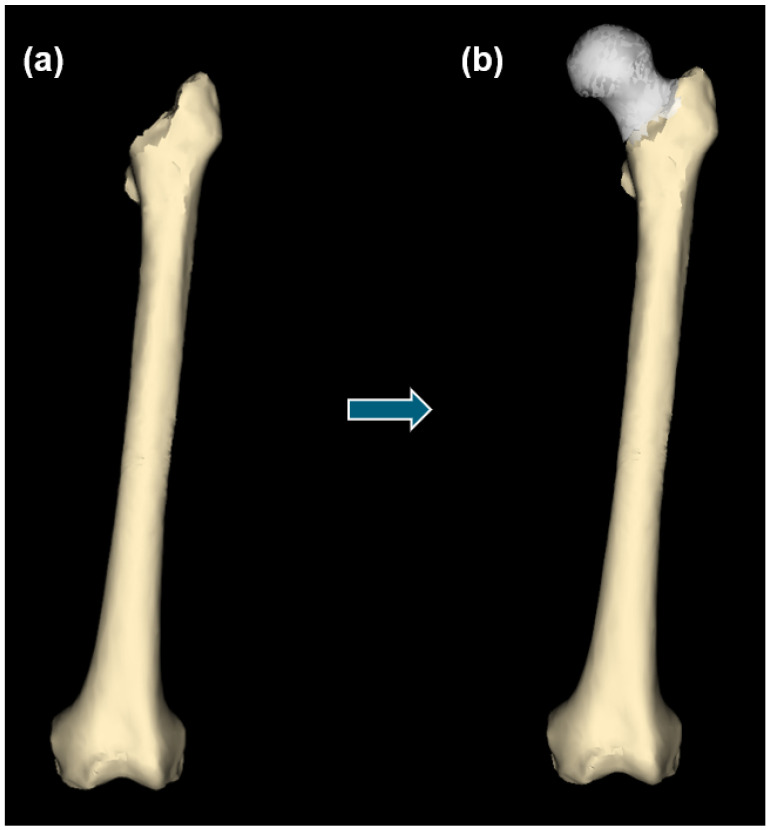
The 3D models of damaged anatomy and patient-specific statistical reconstructions of healthy anatomical structure: (**a**) damaged left femur; (**b**) whole left femur after statistical reconstruction of missing structure. Bone is shown in beige, and statistically reconstructed anatomy is shown in transparent white; anterior view.

The maximum femoral length data showed clear differences between the femurs with traditional prostheses and the statistically reconstructed femurs. The traditionally repaired femurs had maximum femoral lengths that were more than 22 mm smaller than the respective patient-specific statistical reconstruction mean structures (Figure 5). The femurs with traditional prostheses had maximum femur lengths that were smaller than all the patient-specific statistical reconstructions, which was consistent with expectations for the maximum femoral length given the traditional prosthetic design (Table 1). The maximum femoral length values of the statistically reconstructed healthy femurs from −3 to +3 standard deviations showed a gradual increase.

Among the statistical reconstructions of the healthy anatomy, the +/−1 SD statistical reconstructions had Hausdorff distances of approximately 2.9 mm. Considerable differences were evident between the Hausdorff distances of the statistically reconstructed anatomical structures and the femur structures with traditional prostheses (Table 2). The femur structures with traditional prostheses had Hausdorff distances that were greater than those of all the statistical reconstructions. For instance, the femur structures with traditional prostheses had Hausdorff distance values that were over 12mm greater than those of the +/−3 standard deviation statistical reconstructions.

The femoral neck anteversion data of femur structures with traditional prostheses displayed femoral neck anteversion values that were much greater than values of all the statistical reconstructions of the healthy structures (Table 3). In particular, the femurs with traditional prosthesis structures were more than nine degrees greater than the most similar statistical reconstructions, whereas the range in the statistical reconstruction femoral neck anteversion values from −3 standard deviation to +3 standard deviation was about one degree. Of the statistically reconstructed healthy femur structures, the femoral neck anteversion values slightly decreased from −3 standard deviation to +3 standard deviation.

The data from the length of the rotational center divergence had values for femur structures with traditional prostheses that were considerably greater than those of all the statistical reconstructions of the healthy anatomy (Table 4). The +/−3 standard deviation statistical reconstructions had lengths of the rotational center divergence of roughly 7.5 mm. Yet, the values for femur structures with traditional prostheses were over 16mm greater than the statistically reconstructed healthy anatomy of +/−3 standard deviations.

The angle of inclination data also indicated clear differences between the femur with traditional hip prosthesis structures and the statistical reconstructions of healthy femur structures (Table 5). The values of the angle of inclination increased from −3 standard deviation to +3 standard deviation for the statistical reconstructions of healthy structure. The angle of inclination values from the femur with traditional prosthesis structures were outside the maximum and minimum values of the statistical reconstructions but were relatively close to the −3 standard deviation statistical reconstruction values. These data for the angle of inclination showed that the femurs repaired with traditional prostheses had values over five degrees smaller than the respective patient-specific statistical reconstruction means.

**Figure 6:**
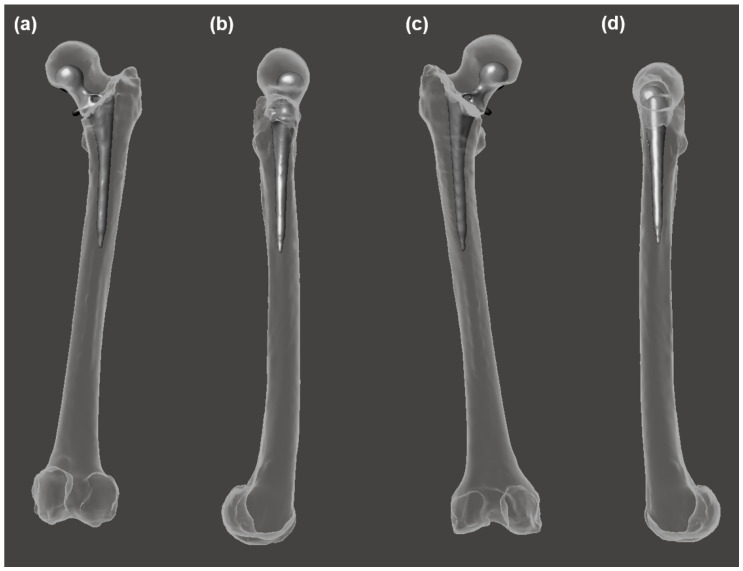
Comparison of the statistical reconstruction of healthy structure and the traditional prosthesis morphology and orientation for the same individual. Prosthesis is shown in gray; bone and statistically reconstructed bone are transparent: (**a**) anterior view; (**b**) medial view; (**c**) posterior view; (**d**) lateral view.

In addition to the marked differences displayed in these quantitative measurements of morphology between the femur structure after traditional hip arthroplasty and the statistical reconstructions of healthy femur structure, the 3D anatomical models revealed some valuable qualitative insights. The surface boundary of the traditional prosthetic extended beyond the surface of the mean statistical reconstruction of the healthy anatomy. This disparity occurred at the inferior surface of the femoral head and near the base of the femoral neck (Figure 6). Additionally, an examination of the 3D anatomical models indicated that prosthesis positioning was offset from the mean statistical reconstruction. The artificial femoral head and neck were positioned along the anterior of the statistically reconstructed healthy structure’s femoral head and neck, rather than being positioned at a central location within the healthy femur structure surface boundaries.

## 4. Discussion

**Figure 7:**
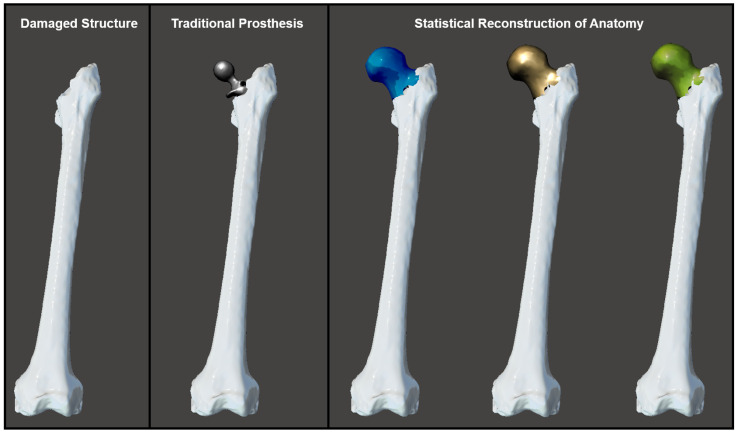
Morphological variation between statistically reconstructed healthy anatomy and that of a traditional prosthesis. Bone is shown in white. Prosthesis is shown in gray. Statistical reconstructions: Mean is shown in gold, −1 SD is shown in blue, and +1 SD is shown in green. SD = standard deviation.

Overall, the results support the hypothesis that femurs with statistical reconstructions of the healthy structure are morphologically distinct from the respective femurs with traditional prostheses (Figure 7). The differences in the maximum femoral length and Hausdorff distance between femurs with prostheses and statistically reconstructed healthy femurs were consistent with expectations given the disparities between the prosthetic femoral head diameter and the natural femoral head diameter. The selection of a smaller femoral head than that occurring in nature for this prosthesis design was based on the strategy that a reduction in the femoral head diameter, and thus its surface area, would decrease the amount of wear [11,16]. Whether or not the femoral head diameter actually changes the wear rate in total hip arthroplasty has been a subject of debate for years, but more recent results suggest that there is no significant difference in the wear rates of prostheses with small femoral heads and prostheses with large femoral heads [24,51].

Regardless of the influence of the femoral head diameter on the wear rate of the prosthesis, there are other functional considerations related to the femoral head diameter. A reduction in the femoral head diameter decreases the femoral head-to-neck ratio, decreases the range of motion in all directions, and increases the chance of impingement, which ultimately increases the likelihood of dislocation [24,52,53]. Moreover, smaller diameter femoral heads, when compared with larger femoral head diameters, require relatively less translation of the femoral head from the acetabulum for dislocation to take place [53]. These principles are directly supported by the results indicating that a greater risk of dislocation was found when smaller femoral heads were used in total hip arthroplasty when compared with the use of larger femoral heads [54,55]. While the functional consequences of femoral head diameter are beyond the scope of the current investigation, the present results do illustrate the dramatic morphological disparity between these prostheses and the healthy anatomy considering that the maximum femoral length and Hausdorff distance values of the prostheses fell outside the values of the statistically reconstructed healthy structures of +3 and −3 standard deviations.

Unlike the maximum femoral length and the Hausdorff distance, the other measurements (i.e., the femoral neck anteversion, the length of the rotational center divergence, and the angle of inclination) are independent of the femoral head diameter. These three types of data should have no difference between the femurs with prostheses and the statistically reconstructed healthy femurs, if the hip arthroplasties accurately recreated these anatomical attributes of healthy femurs. Interestingly, the results from the femoral neck anteversion, the length of the rotational center divergence, and the angle of inclination indicate substantial differences between the femurs with prostheses and the statistical reconstructions of the healthy anatomy. The values from femurs with prostheses did not fall within the values of the +/−3 standard deviation statistical reconstructions.

The disparities in the lengths of the rotational center divergence of the femurs with hip prostheses and the statistical reconstructions are especially intriguing and not without consequence. During hip arthroplasty, it is crucial to accurately restore the center of rotation in order to preserve biomechanical function [56]. The incorrect positioning of the center of rotation is a factor that increases the probability of dislocation [9]. Poor positioning of a prosthesis can negatively influence patients, either through a loss of biomechanical function in the hip joint or by increasing the probability of the prosthetic device failing entirely [57]. A greater risk of dislocation has been found in arthroplasties that have been performed by less experienced surgeons [58]. Upon consideration of these factors, the results suggest that the prostheses examined in this study may not have been accurately positioned during surgery to preserve the natural biomechanical function of these patients.

Similarly, the 3D anatomical models of the statistical reconstructions revealed additional information indicative of the incorrect positioning of the prostheses on these damaged femurs. Each of the prostheses exhibited a slight tilt on the longitudinal axis that contributed to an anterior projection of the prosthetic femoral head and neck relative to the femoral head and neck of the healthy structure. Furthermore, the femoral stem of each prosthesis was not centered on the longitudinal axis of the femoral shaft (Figure 6). The correct positioning of a hip prosthesis occurred when the femoral stem was centered within the shaft of the femur [9]. The offset positioning of the prosthesis in each of these cases may be due to deliberate decisions by the surgeons based on challenges during surgeries, such as the need to limit the amount of time the patient was under anesthesia or difficulties anchoring the prosthesis to the damaged femur. Nonetheless, these findings reveal not only that the morphology differs between the femurs with prostheses and the healthy femur statistical reconstructions, but that the prosthesis positioning was not ideal for these patients with regard to ensuring the success of the implant or maintaining the natural biomechanical function.

The successful treatment of hip fracture and pathology is critical for avoiding increased demands on the healthcare system, reoperation, loss of independence, development of physical disability, and death of patients [12]. The quantitative morphological data from the current study are consistent with the findings of previous research, which showed that traditional hip prostheses need to be improved in order to prevent negative outcomes for patients [1,9,11,23,24,32]. The incorporation of patient-specific statistical reconstructions of healthy anatomical structures into hip prosthesis development and surgical planning could help to ensure that patients have positive outcomes following hip arthroplasty.

The present study has the limitation that it only demonstrates this new approach using the model system of hip prostheses. Quantitative morphological differences were evident between the femurs repaired with traditional hip prostheses and the patient-specific statistical reconstructions of the healthy femur structure, but morphological differences may not be as significant between other anatomical structures repaired with prostheses and statistical reconstructions of those respective healthy structures. Therefore, although patient-specific statistical reconstructions have the potential to improve many prosthesis designs and surgical procedures, the results of the current study only suggest that hip prostheses are in need of further improvement.

## 5. Conclusions

Extensive study of the modern hip joint prosthesis showed that there is a critical need for subject-specific data to inform medical implant development so that implant failure is reduced [26,37]. The current study describes a new approach in which subject-specific information from damaged femur structure is used to statistically reconstruct subjects’ healthy anatomical structures. Although further research is needed to determine the extent to which other types of medical implants besides hip prostheses need to be improved, this new computational technique can potentially be applied to treating patients with injuries or pathologies afflicting a variety of anatomical structures.

A CT scan of the patient’s damaged anatomy in combination with a SSM generated from the particular healthy anatomical structure could be used to create a patient-specific statistical reconstruction of the missing healthy structure, which has many positive potential applications. Generating patient-specific statistical reconstructions of the healthy structure might be helpful for informing prosthetic design so that the prostheses more closely resemble natural anatomical structures. Furthermore, someday, this concept could be used to generate 3D-printed custom prostheses for patients. These new prostheses could potentially reduce recovery time, have lower risks of failure, and more effectively restore natural biomechanical function for the patient.

This approach could also be applied in the surgical setting by using diagnostic CT data to rapidly provide surgeons with a visual guide. The statistical reconstructions of a patient’s anatomy could inform prosthetic device selection and support more accurate positioning of the prosthesis. All in all, the statistical reconstruction of patient-specific anatomy has the potential to augment prosthetic device design and aid surgical procedures, thereby decreasing the likelihood of prosthetic implant failure, decreasing recovery time, and more effectively restoring a patient’s natural biomechanical capabilities.

## Figures and Tables

**Table 1:** Maximum femoral length (mm) data of femur structures with traditional prostheses and healthy femur structures obtained by statistical reconstruction. TrPr = traditional prosthesis; StatRecon = statistical reconstructions; SD = standard deviation.

	TrPr	StatRecon	StatRecon	StatRecon	StatRecon	StatRecon	StatRecon	StatRecon
		−3SD	−2SD	−1SD	Mean	+1SD	+2SD	+3SD
PASWSRA	461.3	475.7	478.1	480.7	483.4	486.2	488.7	491.6
PASWSRB	439.5	454.8	457.3	459.9	462.5	465.2	467.8	470.6

**Table 2:** Hausdorff distance (mm) data of femur structures with traditional prostheses and healthy femur structures obtained by statistical reconstruction. All values represent the Hausdorff distance between the particular specimen and the respective statistical reconstruction mean. TrPr = traditional prosthesis; StatRecon = statistical reconstruction; SD = standard deviation.

	TrPr	StatRecon	StatRecon	StatRecon	StatRecon	StatRecon	StatRecon
		−3SD	−2SD	−1SD	+1SD	+2SD	+3SD
PASWSRA	25.5	8.5	5.7	2.9	2.9	5.9	8.8
PASWSRB	29.0	8.4	5.7	2.8	2.9	5.7	8.6

**Table 3:** Femoral neck anteversion (°) data of femur structures with traditional prostheses and healthy femur structures obtained by statistical reconstruction. TrPr = traditional prosthesis; StatRecon = statistical reconstructions; SD = standard deviation.

	TrPr	StatRecon	StatRecon	StatRecon	StatRecon	StatRecon	StatRecon	StatRecon
		−3SD	−2SD	−1SD	Mean	+1SD	+2SD	+3SD
PASWSRA	17.2	6.2	6.1	6.0	5.8	5.6	5.5	5.4
PASWSRB	13.0	4.5	4.4	4.3	3.9	3.8	3.7	3.6

**Table 4:** Length of rotational center divergence (mm) data of femur structures with traditional prostheses and healthy femur structures obtained by statistical reconstruction. All values represent length of rotational center divergence between the particular specimen and the respective statistical reconstruction mean. TrPr = traditional prosthesis; StatRecon = statistical reconstructions; SD = standard deviation.

	TrPr	StatRecon	StatRecon	StatRecon	StatRecon	StatRecon	StatRecon
		−3SD	−2SD	−1SD	+1SD	+2SD	+3SD
PASWSRA	21.1	7.0	4.7	2.2	1.6	4.6	8.3
PASWSRB	16.6	7.4	5.5	2.8	3.0	4.9	8.6

**Table 5:** Angle of inclination (°) data of femur structures with traditional prostheses and healthy femur structures obtained by statistical reconstruction. TrPr = traditional prosthesis; StatRecon = statistical reconstructions; SD = standard deviation.

	TrPr	StatRecon	StatRecon	StatRecon	StatRecon	StatRecon	StatRecon	StatRecon
		−3SD	−2SD	−1SD	Mean	+1SD	+2SD	+3SD
PASWSRA	129.3	129.8	132.7	134.6	135.7	137.9	139.2	142.2
PASWSRB	128.1	131.4	132.5	133.8	135.0	136.4	137.9	138.5

## Data Availability

The data are contained within the article.

## Abbreviations

The following abbreviations are used in this manuscript:

CT	Computed Tomography
SSM	Statistical Shape Model
UB	University at Buffalo
CTRC	Clinical and Translational Research Center
ICP	Iterative Closest Points
PCA	Principal Component Analysis
GP	Gaussian Process
SD	Standard Deviation
StatRecon	Statistical Reconstructions
FNA	Femoral Neck Anteversion
NSA	Neck-Shaft Angle
TrPr	Traditional Prosthesis
